# Cracking the Case: Post-pregnancy Bilateral Femoral Fragility, a Rare Clinical Challenge

**DOI:** 10.7759/cureus.82986

**Published:** 2025-04-25

**Authors:** Jijisha Ali, Janaki Gopalan, Youssef Fallaha, Shriganesh Patil, Rida M Maryum

**Affiliations:** 1 Department of Obstetrics and Gynaecology, Mediclinic Welcare Hospital, Dubai, ARE; 2 Department of Orthopedic Surgery, Mediclinic Welcare Hospital, Dubai, ARE; 3 Department of Radiology, Mediclinic Welcare Hospital, Dubai, ARE; 4 College of Medicine, Mohammed Bin Rashid University of Medicine and Health Sciences, Dubai, ARE

**Keywords:** avascular necrosis, bilateral hip insufficiency fractures, femoral fragility, pregnancy-associated musculoskeletal disorders, pregnancy-associated osteoporosis

## Abstract

Pregnancy-associated musculoskeletal disorders are relatively rare but can present significant diagnostic and therapeutic challenges. We present a rare case of a 37-year-old woman with a twin pregnancy complicated by type 2 diabetes mellitus and obstetric cholestasis, who developed bilateral hip insufficiency fractures. Despite initial conservative management, her condition worsened, requiring surgical intervention in the form of core decompression and internal fixation. Postoperative recovery was successful, with significant pain relief and improved mobility. This case underscores the complexity of pregnancy-related musculoskeletal disorders and the need for timely diagnosis and appropriate treatment, especially in high-risk pregnancies.

## Introduction

Pregnancy-associated musculoskeletal disorders are uncommon but can lead to significant morbidity, especially in high-risk pregnancies. These conditions encompass a spectrum of disorders, including sacroiliitis, avascular necrosis (AVN), stress fractures, and transient osteoporosis, all of which can be debilitating if not diagnosed and managed appropriately [1]. The physiological changes of pregnancy, such as increased joint laxity due to relaxin, weight gain, and altered biomechanics, contribute to an increased risk of musculoskeletal complications, particularly in women with underlying metabolic conditions [2].

AVN of the femoral head is a rare but serious condition in pregnancy, often attributed to hormonal and vascular changes that predispose patients to bone ischemia [3]. The pathogenesis of AVN involves osteocyte and bone marrow cell death as a result of blood flow disturbance to the afflicted femoral head, which ultimately causes necrosis and subchondral bone collapse [4]. Hip osteonecrosis has been associated with a range of etiological factors, including trauma, alcohol misuse, high doses of corticosteroid therapy, genetic susceptibility, coagulation problems, and sickle cell disease. Additionally, sacroiliitis, though often associated with seronegative spondyloarthropathies, can occur during pregnancy due to increased mechanical stress on the sacroiliac joints [5]. Insufficiency fractures of the femoral neck, while exceptionally rare, may develop due to transient osteoporosis or mechanical overload, particularly in the context of gestational diabetes or other metabolic disorders. Pregnancy-associated osteoporosis (PAO) is a rare condition characterized by fragility fractures that typically occur non-traumatically during late pregnancy or lactation, placing the maternal skeleton at risk. During these periods, the maternal skeleton undergoes substantial alterations in calcium and bone metabolism to support fetal development and lactation, potentially increasing the risk of insufficiency fractures. However, PAO is not fully understood, and its exact causes are thought to involve genetic predisposition, hormonal changes, and nutritional factors. The most common manifestation of PAO during pregnancy is vertebral fracture, whereas femoral neck fractures are considered rare complications [6]. Women with pre-existing low bone mineral density (BMD), due to underlying conditions affecting bone mass, may face an increased risk of fractures in the later stages of pregnancy.

Pregnancy is not a known risk factor for femoral head AVN, and it should be distinguished from transitory hip osteoporosis, one of the more prevalent hip diseases during pregnancy. Even in otherwise healthy individuals, transient osteoporosis of pregnancy (TOP) can lead to bone marrow edema and demineralization, thereby increasing the risk of fracture in the absence of significant trauma. Similarly, AVN may occur during pregnancy in women without identifiable risk factors. If left untreated, both transient osteoporosis and AVN arising during pregnancy can progress beyond the postpartum period and may lead to long-term complications [7].

## Case presentation

A 37-year-old woman, gravida 2, para 1, conceived spontaneously and was booked for antenatal care at six weeks of gestation. Her medical history included type 2 diabetes mellitus (T2DM), which was managed with insulin, and a history of obstetric cholestasis, for which she was treated with ursodeoxycholic acid from 20 weeks of gestation. Her pregnancy progressed uneventfully until 22 weeks when she developed pubic symphysis pain, prompting a referral to orthopedics. Initial ultrasound and clinical examination revealed no soft tissue masses, abnormal vascularity, or focal collections. Despite initiating physiotherapy, her symptoms worsened and spread to both hips. By 24 weeks, she required a walker at home and a wheelchair in the hospital. A neurological assessment suggested sacroiliac joint pain with possible lateral cutaneous nerve entrapment.

At 35+3 weeks, an elective lower-segment cesarean section (LSCS) was performed due to intrauterine growth restriction (IUGR) of the second twin. Postnatally, her condition deteriorated, with worsening bilateral thigh pain, weakness, and difficulty walking. A repeat orthopedic referral was made for further evaluation. Postnatal imaging studies were conducted to identify the cause of her symptoms.

**Figure 1:**
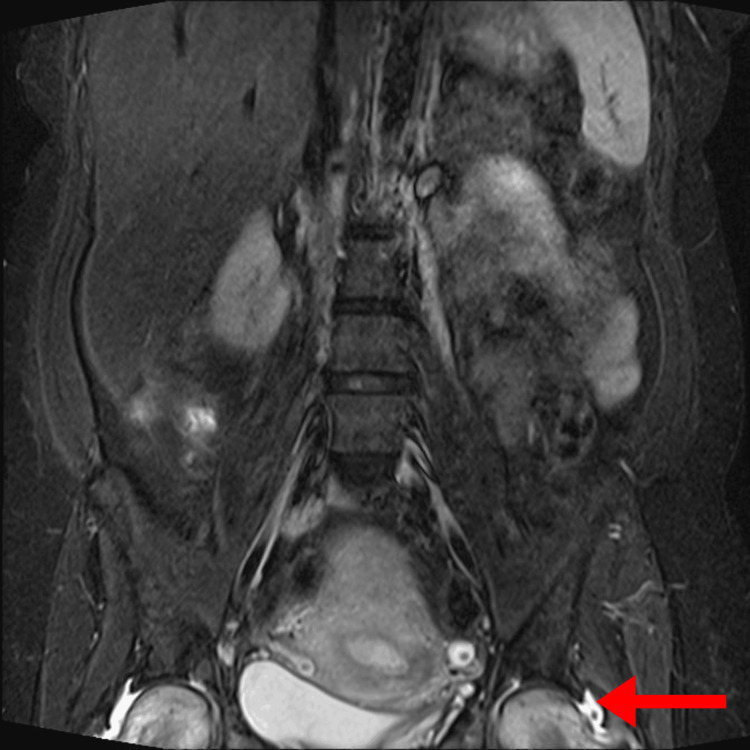
MRI of the lumbosacral spine showing bilateral femoral head STIR hyperintensities MRI, magnetic resonance imaging; STIR, short tau inversion recovery.

**Figure 2:**
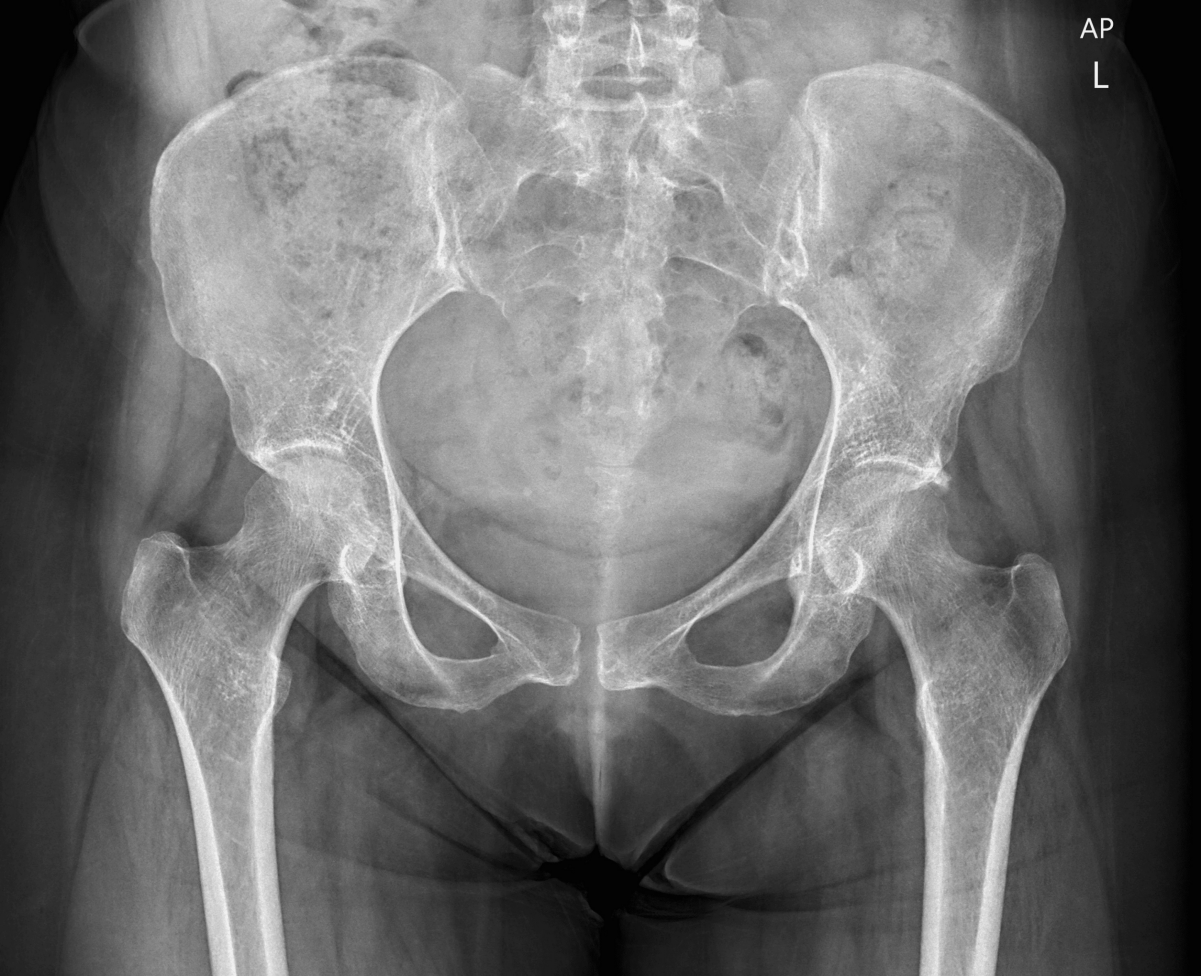
Pelvic X-ray showing no obvious subchondral or subcapital fracture lines

**Figure 3:**
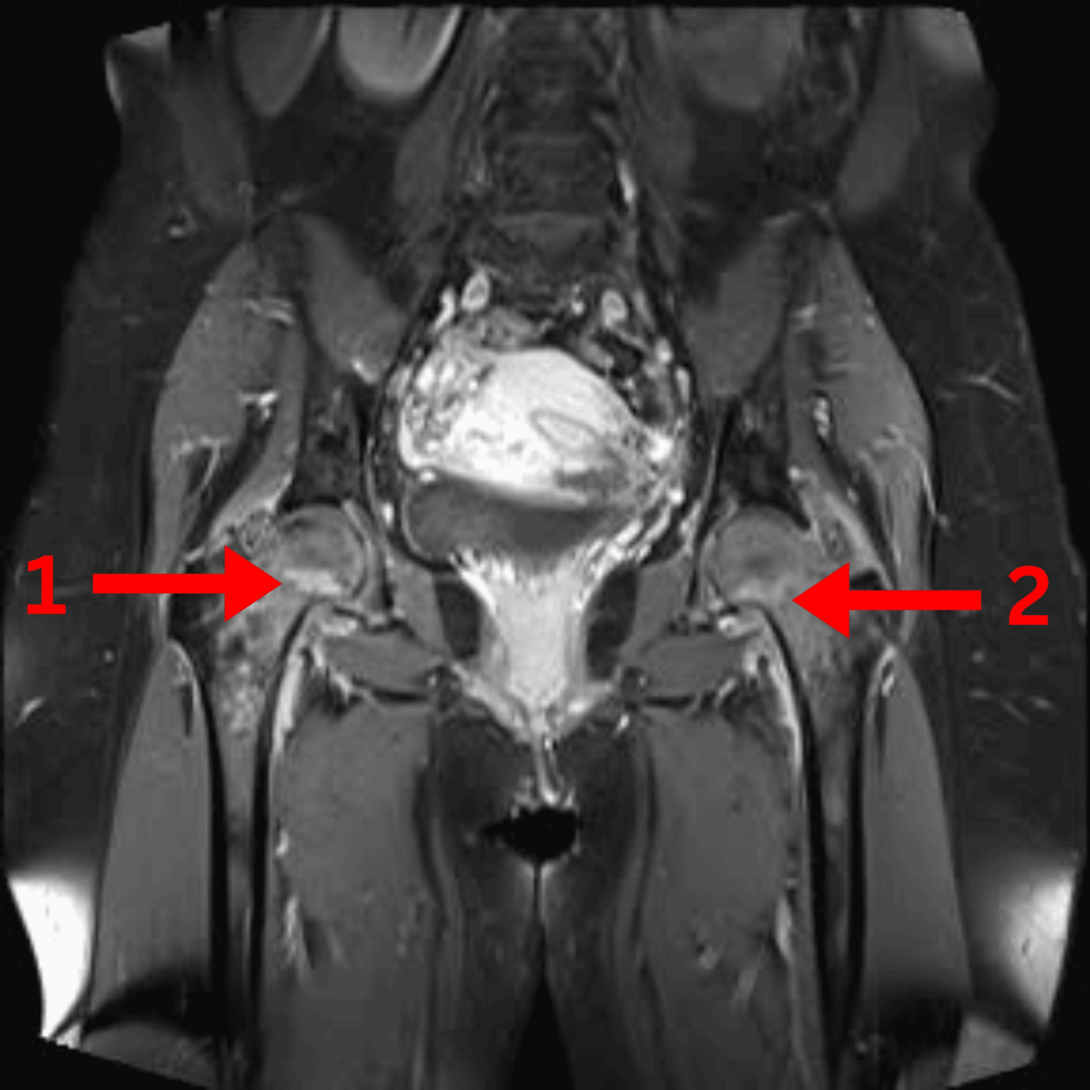
MRI of the hips with contrast showing (1) synovitis and (2) a fracture line MRI, magnetic resonance imaging.

**Figure 4:**
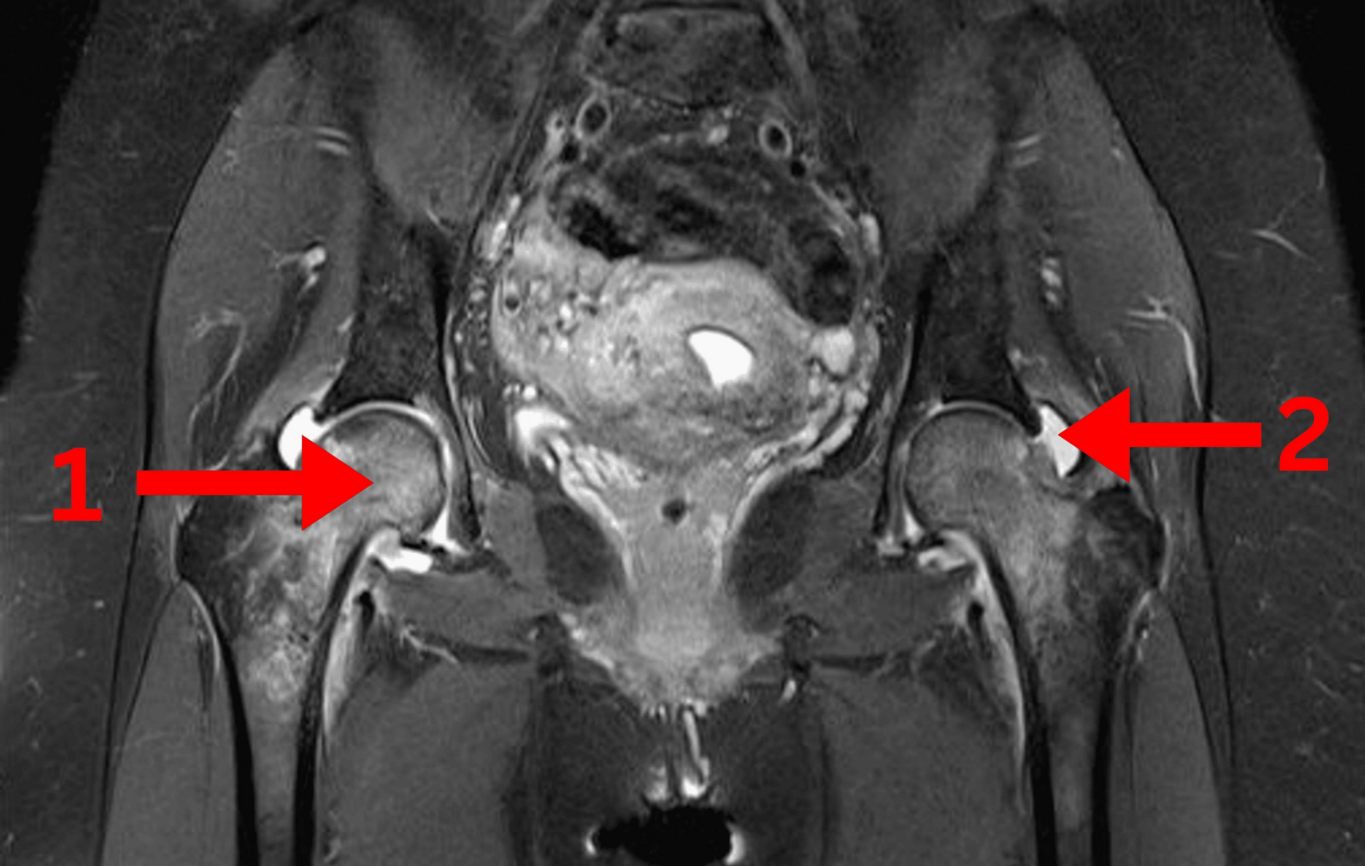
MRI of the hips without contrast showing (1) STIR bright signals and (2) hip joint effusion MRI, magnetic resonance imaging; STIR, short tau inversion recovery.

**Figure 5:**
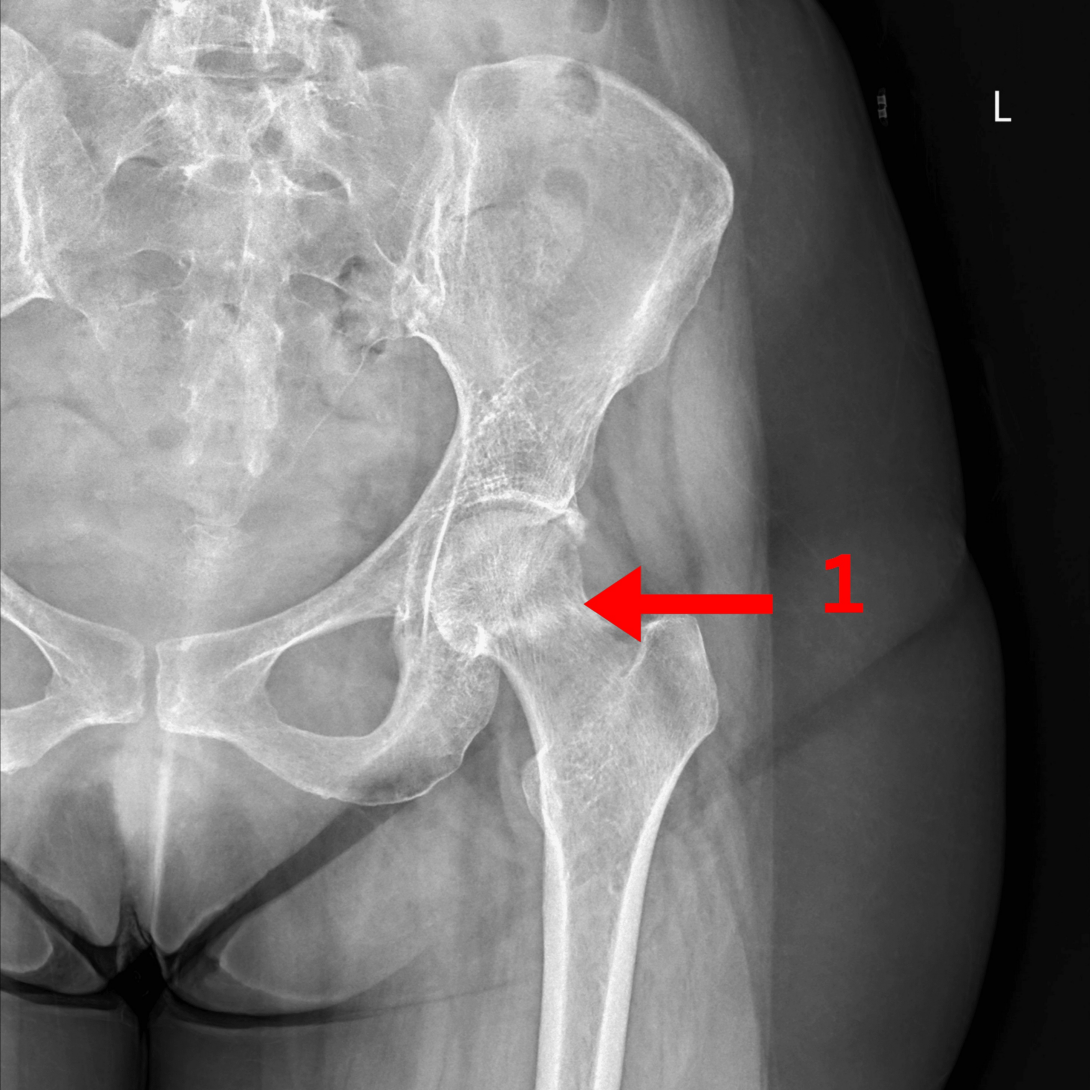
X-ray of the left hip showing (1) subcapital sclerotic fracture line

Imaging studies revealed several findings. Magnetic resonance imaging (MRI) of the lumbosacral spine (Figure 1) showed mild lumbar degenerative disc disease, right-sided sacroiliitis, bilateral hip joint effusion, synovitis, and hyperintensities in both femoral heads, suggestive of transient osteoporosis or early AVN. Pelvic X-rays (Figure 2) revealed normal findings. MRI of the hips with (Figure 3) and without contrast (Figure 4) demonstrated thin short tau inversion recovery (STIR) hyperintensities at both femoral necks, indicative of insufficiency fractures, along with heterogeneous T2-weighted hyperintensities in both femoral heads and necks, raising suspicion of early AVN. Additionally, bilateral sacroiliitis, gluteus medius tendinitis, and trochanteric bursitis were observed, along with mild-to-moderate hip joint effusion and synovial thickening. X-ray of the left hip (Figure 5) revealed fragility fractures at the base of the femoral heads extending to the neck.

Given the patient's clinical presentation and imaging findings, the differential diagnosis included TOP, early AVN, and stress fractures. TOP was considered due to its self-limiting nature and resolution postpartum. Early AVN was also considered, given the patient's T2DM, which can compromise femoral head perfusion. However, the absence of a distinct serpiginous subchondral signal change on MRI made AVN less likely. Stress fractures were suspected, especially given the fragility fractures extending from the base of the femoral heads to the neck, which suggested a more severe pathology. Following discussion between the orthopedic and radiology teams, the most consistent diagnosis was determined to be bilateral fragility fractures at the base of the femoral heads extending to the neck, with associated bone bruising.

**Figure 6:**
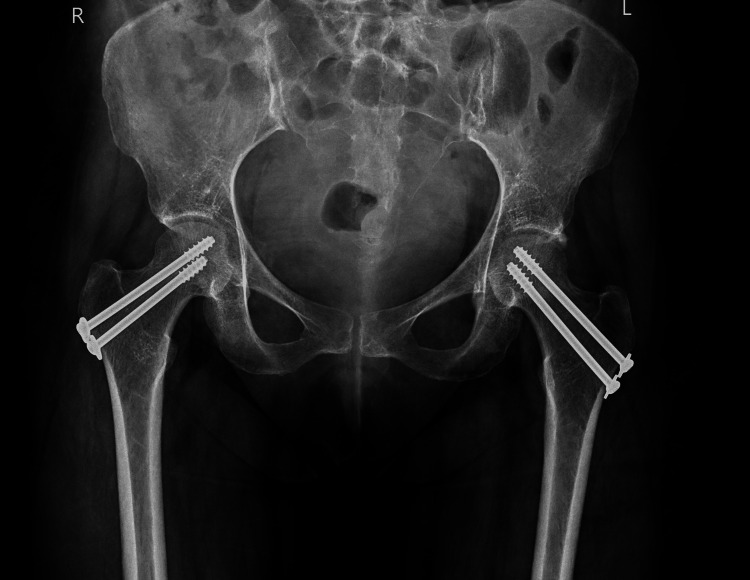
Postoperative X-ray

Initial management involved physiotherapy and analgesia, which provided limited relief. Bilateral sacroiliac joint injections with bupivacaine and Depo-Medrol were administered, but did not significantly alleviate her symptoms. Given the progressive nature of her condition and the high risk of femoral head collapse, surgical intervention was deemed necessary. The patient underwent bilateral core decompression and internal fixation with cannulated screws to stabilize the fractures and preserve joint integrity. The procedure involved the placement of guidewires under image intensification, followed by drilling with a 4.5 mm drill and insertion of 7.5 inch cannulated screws in parallel on the anteroposterior (AP) view, angled laterally for optimal stabilization. Postoperative X-rays (Figure 6) confirmed proper screw placement, with preserved hip joint space and normal femoral head contours.

Postoperatively, the patient underwent intensive physiotherapy, which resulted in significant pain relief and gradual improvement in mobility. She transitioned from being wheelchair-dependent to ambulating independently. Over time, her sacroiliitis and gluteus medius tendinitis resolved. Follow-up radiographs confirmed stable fixation with no further episodes of hip pain, and the patient continued to show progress in her recovery.

## Discussion

Pregnancy-related musculoskeletal complications, such as insufficiency fractures, are rare but can be potentially debilitating and can present significant diagnostic and therapeutic challenges. This case of a 37-year-old woman with twin pregnancy developing bilateral femoral fragility post-pregnancy underscores the importance of early diagnosis and appropriate imaging in patients with severe musculoskeletal pain during pregnancy. The etiology of pregnancy-associated AVN and transient osteoporosis of the hip (TOH) is multifactorial. Hormonal changes during pregnancy, particularly elevated levels of relaxin and progesterone, lead to increased joint laxity, predisposing joints to instability and microtrauma [8]. Additionally, a higher incidence of hip AVN has been associated with certain endocrine alterations that raise unbound cortisol levels during pregnancy. Additionally, parathyroid hyperplasia can happen during pregnancy, elevating parathyroid hormone levels and raising the risk of osteonecrosis [9].

The gravid uterus alters maternal biomechanics, increasing stress on the hip and pelvic joints. In this patient, a history of T2DM managed with insulin may have further contributed, as diabetes is a known risk factor for microvascular complications that can impair blood supply to the femoral head. Additionally, ovulation stimulation, known to activate coagulation and fibrinolytic pathways, has been proposed as a potential contributing factor [10]. However, our patient conceived spontaneously with no history of ovulation induction.

Vascular compromise may also result from mechanical compression during pregnancy, with reports suggesting that damage to the artery within the round ligament or femoral region may occur following difficult labor or uterine expansion. During the third trimester, when fetal skeletal mineralization accelerates, approximately 80% of the calcium required by term is transferred from the mother. This demand is typically met by enhanced maternal intestinal calcium absorption; however, in cases of inadequate intake, maternal bone resorption may occur. Such resorption, coupled with increased maternal weight, can place additional stress on weight-bearing joints. In our case, the patient’s serum calcium level was within normal limits [11].

While alcohol consumption and corticosteroid use are the most common causes of atraumatic osteonecrosis of the femoral head [12], other risk factors include genetic predisposition, vasculitis, hypercoagulable states, cocaine use, and microemboli formation [13]. Approximately 20% of cases are classified as idiopathic, and osteonecrosis of the femoral head remains a rare complication of pregnancy [14].

Accurately diagnosing hip pain during pregnancy is crucial for patient care. Although AVN and transitory hip osteoporosis appear somewhat alike on MRI, the clinical results differ significantly. The third trimester is when TOH most frequently occurs. It is a self-limiting disorder that gradually goes away over the course of six to nine months. However, the majority of cases of hip AVN necessitate surgery and have a sneaky onset with steadily increasing discomfort. Additionally, AVN is far less prevalent than temporary hip osteoporosis during pregnancy [15].

Several cases of pregnancy-associated AVN and transient osteoporosis have been previously reported. In a case series by Ugwonali et al. (2008), four pregnant women developed hip pain during the third trimester, later diagnosed as AVN. Three required core decompression, while one recovered with conservative management [16]. Toussia-Cohen et al. (2023) conducted a retrospective analysis on all women who had been diagnosed with unilateral or bilateral transient hip osteoporosis by MRI during pregnancy or the nine years following delivery. According to their research, women diagnosed with TOH were typically older, had a low body mass index, a positive family history of osteoporosis, a history of smoking, and had undergone in vitro fertilization (IVF) [17]. A similar case was reported by Mouchantaf et al. (2021), who observed a higher incidence of pregnancy-related AVN in twin pregnancies, likely attributed to increased mechanical and metabolic stress [18]. Evidence suggests that pregnancy-related AVN of the femoral head is more frequently observed in twin gestations, likely due to increased mechanical and metabolic demands. Notably, our patient was carrying twins.

Given the progressive nature of AVN and the potential for irreversible joint damage, early recognition and intervention are critical. Imaging modalities such as MRI are essential for early diagnosis, particularly in cases with persistent pain despite conservative management. When assessing hip pain during pregnancy, MRI is the most accurate method since it can identify abnormalities as soon as 48 hours after the onset of symptoms [19]. Hip diseases can be distinguished from one another according to signal changes visible on T1- and T2-weighted imaging. On T2-weighted images, for instance, transitory osteoporosis of the hip usually manifests as a widespread rise in signal intensity, whereas localized alterations and the pathognomonic double-line sign are characteristics of AVN. Additionally, a fracture line on an MRI makes it easy to identify femoral neck stress fractures [20].

The coexistence of fragility fractures at the base of the femoral heads extending to the neck and AVN of the hip presents a rare and complex clinical scenario. Fragility fractures, often associated with osteoporotic or metabolic bone disease, occur due to reduced bone strength and mechanical insufficiency, whereas AVN results from compromised vascular supply, leading to ischemic necrosis and subsequent femoral head collapse. The presence of both conditions in a single patient complicates diagnosis, prognosis, and treatment planning, requiring a multidisciplinary approach. One of the primary challenges in such cases is determining the primary pathology - whether the AVN predisposed the bone to fracture or whether the fracture initiated the ischemic process leading to AVN.

From a treatment perspective, the management goals involve preventing femoral head collapse, preserving joint function, and addressing biomechanical instability. While core decompression and bone grafting may be effective in early-stage AVN, the presence of a fragility fracture often necessitates cannulated screw fixation to provide structural stability. However, surgical interventions must be carefully planned to avoid compromising the remaining vascular supply, which could exacerbate necrosis and lead to total joint failure. Additionally, patient factors such as underlying metabolic disorders (e.g., diabetes, osteoporosis), pregnancy-related hormonal changes, and biomechanical stress from increased body weight must be taken into account, as they can influence fracture healing and AVN progression. Given the rarity and complexity of concurrent fragility fractures and AVN of the hip, individualized treatment strategies guided by orthopedic, radiology, and metabolic specialists are essential to optimize outcomes and prevent long-term disability. This case emphasizes the significance of diagnosing AVN during pregnancy at an early stage to prevent severe consequences. Although serious hip pathology is uncommon during pregnancy, MRI should be considered without hesitation when clinically indicated.

## Conclusions

This case highlights the importance of considering insufficiency fractures in pregnant patients presenting with persistent musculoskeletal pain. The progression of symptoms despite conservative management emphasizes the need for early recognition and a multidisciplinary approach to ensure timely diagnosis and appropriate treatment. This case also reinforces the significance of thorough orthopedic evaluation in pregnant patients presenting with persistent musculoskeletal pain, as well as the need for appropriate imaging when conservative measures fail. Ultimately, timely surgical intervention, including core decompression and internal fixation, proved essential in preventing further joint collapse and restoring mobility. Future studies should explore the underlying pathophysiological mechanisms and optimal management approaches for AVN and insufficiency fractures in pregnancy to improve outcomes for affected patients.
